# Explore the effects of forest travel activities on university students’ stress affection

**DOI:** 10.3389/fpsyg.2023.1240499

**Published:** 2024-01-10

**Authors:** Wei-Yin Chang, Xin Wang, De-Sheng Guo, Lam-Huu-Phuoc Nguyen, Ngoc-Huy Tran, Shuai-Jie Yang, Hui-Zhong Lin, Hsiu-Chen Wu, Chin-Fei Huang

**Affiliations:** ^1^College of Forestry, Fujian Agriculture and Forestry University, Fujian, China; ^2^Graduate Institute of Science Education and Environmental Education, National Kaohsiung Normal University, Kaohsiung, Taiwan; ^3^Department of Cardiology, Fujian Medical University Union Hospital, Fuzhou, China; ^4^Department of Food and Beverage Management, Cheng Shiu University, Kaohsiung, Taiwan

**Keywords:** forest travel, physiological health, psychological health, stress affection, university student

## Abstract

This study aims to explore the effects of forest travel activities on university students’ stress affection. Forty volunteer university students participated in this study. All participants were asked to complete physiological (Heart Rate Variability) and psychological (Brief Profile of Mood State and State–Trait Anxiety Inventory) tests before and after the travel activities. The results reported that students’ heart rates were significantly lower after the forest travel activities than before. All domains of negative mood and anxiety decreased from the pre-test to the post-test. This study found that university students could feel less stressed if they went on forest travel activities.

## 1 Introduction

Stress is a challenge for modern society and a health pandemic of the 21st century (Tsunetsugu et al., 2007; Park et al., 2010; Fink, 2017). This issue has attracted scholars since antiquity because of stress’s detrimental impacts on mental and physical health (Fink, 2017; Ribeiro et al., 2018; Miriam et al., 2021). Nowadays, high-stress levels are a social problem experienced by university students worldwide. The university students felt high pressure not only because of academic pressure or taking tests but also because of environmental learning changes, financial problems, and family expectations (Ribeiro et al., 2018; Kim et al., 2021). Additionally, social issues in the whole world also affect university students’ mental health, such as COVID-19 (Schwartz et al., 2021). Based on previous studies, more than 50% of university students feel anxious and depressed in their daily life, especially in Asian countries (Craggs, 2012; Downs and Eisenberg, 2012). Take China as an example; there were about 7.6 million university graduates in 2018 (National Bureau of Statistics of China, 2019). These numbers mean that one student needs to compete with many other students in their studies through to their working life, which causes them stress in China (Wen et al., 2022; Jiang, 2023). The term “stress” was introduced by Hans Selye, which can be understood as the body’s response to problems or tasks, depending on how the individual controls them (Fink, 2017; Dagnino-Subiabre, 2022). The results of excessive stress can lead to a negative impact on life, such as drug misuse, suicide, insomnia, exhaustion, depression, and reduced academic performance (Shah et al., 2010; Downs and Eisenberg, 2012; Cavallo et al., 2016; Ribeiro et al., 2018). Furthermore, stress is implicated in a significant portion of lifestyle-related diseases, including hypertension and elevated cortisol levels, increasing susceptibility to infections and disrupting glucose tolerance, ultimately contributing to diabetes (Merabet et al., 2022; Sharma et al., 2022). Over time, these health issues may lead to arteriosclerosis, constituting a substantial portion of illnesses and incurring economic damage to the healthcare system (Inoue, 2014). University students are one of society’s future human resources. Strengthening their physical and psychological health is vital for creating the foundation for a healthy future in society. So, universities need strategies or methods to help students feel less stressed.

Among many strategies to decrease stress, exposing students to nature and the green environment is considered one of the most effective ways (Hansen et al., 2017; Liu et al., 2018; Markwell and Gladwin, 2020; Antonelli et al., 2021). A growing number of studies have explored the topic of “forest therapy” and “forest bathing” in enhancing physical and mental health in recent years (Tsunetsugu et al., 2007; Park et al., 2010; Nan et al., 2013; Song et al., 2017; Bielinis et al., 2018; Chen et al., 2018; Hassan et al., 2018; Liu et al., 2018; Lin et al., 2019). Both approaches, “forest therapy” and “forest bathing” involve travel or therapeutic activities in forest environments. They have been found to contribute to reducing anxiety and stress and improving mental health (Park et al., 2010; Song et al., 2015; Bielinis et al., 2018; Chen et al., 2018), but also helped individuals decrease negative feelings (Korpela, 2003; Shin et al., 2012; Chun et al., 2017; Bielinis et al., 2019; Lyu et al., 2019) and improve positive emotions (Ikei et al., 2014; Jung et al., 2015; Sonntag-Öström et al., 2015; Gong et al., 2017; Meyera and Botsch, 2017; Oh et al., 2017; Bielinis et al., 2018; Yau and Loke, 2020; Li et al., 2021). When compared to urban environments, the forest environment helps university students have lower pulse rates and blood pressure, and walking in the forest environment favorably influences cardiovascular responses and helps them reduce stress (Lee et al., 2014). In addition, the profile of mood states (POMS) decreased after the forest experience (Mao et al., 2012a,b). Also, university students were encouraged to frequent the forests frequently, which helps reduce anxiety and depression (Zhuo and Sun, 2014). Although previous studies have provided evidence to illustrate the positive effects of forest travel activities on health, most focused on aging or sick people (Shin et al., 2012; Lee et al., 2014; Sonntag-Öström et al., 2015; Chun et al., 2017; Chen et al., 2018; Rajoo et al., 2020; Kotera and Fido, 2021). There are not currently so many studies investigating whether forest travel activities are beneficial to young people, especially university students.

Forest travel activities are recreational and calming activities in the forest that bring comfort and decrease stress for participants, such as strolling, combined with breathing in the air (Li et al., 2007; Tsunetsugu et al., 2010; Oh et al., 2017; Kil et al., 2021) and observing and listening to the natural world. Forest travel activities are not the same as hiking or physical activity (Yoshinori et al., 1998). Participants experience the forest landscape, the ambiance of the forest, the sounds of the forest, and the fresh air via their senses (Cheng et al., 2021; Li et al., 2021). Participants can obtain a comprehensive awareness of the ecology in this forest and a better appreciation of the diversity of the natural world (Lee et al., 2014). As a result, forest travel improves physical and mental health (Lee et al., 2014; Li et al., 2021). In addition, green spaces on campus help students breathe fresh air, relax, or stroll, which contributes to enhancing their mental health after school and decreasing their stress levels (Ribeiro et al., 2018). However, different forests may have different impacts on stress affection because their trees, concentrations of anions, and phytoncide chemical components are different (Hauru et al., 2012; Norimasa et al., 2014; Yu et al., 2019; Jamali et al., 2020).

In this study, we take forest travel activities as the core concepts to explore their effects on university students’ stress affection. In the southern of China, Lanyuan Forest was selected for this investigation. If the activities in this forest could assist in reducing students’ stress, then this forest or others might become a natural therapeutic place for university students. Whereby, extending this therapy model to other schools with forests attached to the school or organizing for students to have forest travel activities in nearby woods could be considered. Therefore, this study sought to seek the answer to the main question “How might travel activities in the Lanyuan Forest assist university students in coping with stress?.” Based on the main question, the following research question was formulated and tested in this study: Do forest travel activities improve both physiological and psychological health, ultimately reducing stress among university students?

## 2 Materials and methods

### 2.1 Participants

This study was conducted in Fujian, China. The sample size for the statistical comparison between pre-test and post-test was determined based on the following calculation: with a significance level (alpha) of 0.05 and a power (1 - beta) of 0.80, a medium effect size (Cohen’s *d* = 0.50), and a two-tailed *t*-test, the required sample size was estimated using G*Power statistical software. The calculation indicated that a minimum of 34 participants would be needed. Then, the criterion sampling method was used in selecting participants. Recruitment notices were posted throughout the information boards in the university buildings to recruit individuals who met the following criteria: (1) students studying at Fujian Agriculture and Forestry University (FAFU); (2) voluntarily engaging in this research; (3) being mentally healthy with no history of neurological or mental disorders; (4) no diagnosis of a reaction to severe stress and/or depression; and (5) not suffering from drug or alcohol abuse. Based on these criteria, there were 40 volunteer university students (10 males, 30 females, mean age ± S.D. = 20.34 ± 1.43 years old, age range = 19–21 years old) from Fujian Agriculture and Forestry University (FAFU) who agreed to participate in the research. The gender distribution of this study reflects the previous research findings that women were more drawn to self-care experiential activities like forest travel than men (McEwan et al., 2021). It could be regarded to explain the greater number of female students participating than their male counterparts in this study.

Before administering the study, the participants were aware of the study’s goal, methods, risks, and benefits. They signed an informed consent form and were notified that their participation was entirely voluntary and that they could withdraw at any time. In this research, all methods were performed in accordance with the relevant guidelines and regulations. Participants were asked to complete the physiological and psychological tests before and after the forest travel activities. The participant information is given in Table 1.

**Table 1:** Socio-demographic distributions of the experience of Lanyuan Forest Footpath in FAFU.

General information	Items	Number	Percentage (%)
Gender	Male	10	25
	Female	30	75
Discipline	Natural science	14	35
	Social science	26	65
Exercise habit	No	16	40
	Yes	24	60
Smoking habit	No	38	95
	Yes	2	5
Drinking habit	No	36	90
	Yes	4	10

All of the data were collected prior to January 2020 (the most recent data were collected on December 17, 2019), and COVID-19 had no effect on any of the activities or data collection processing. The weather on that day was cloudy without rain. The authors had been trained in the Code of Ethics of the World Medical Association, and the study was approved by the ethics committee of Fujian Agriculture and Forestry University Human Research Ethics Committee.

### 2.2 Study sites

**Figure 1:**
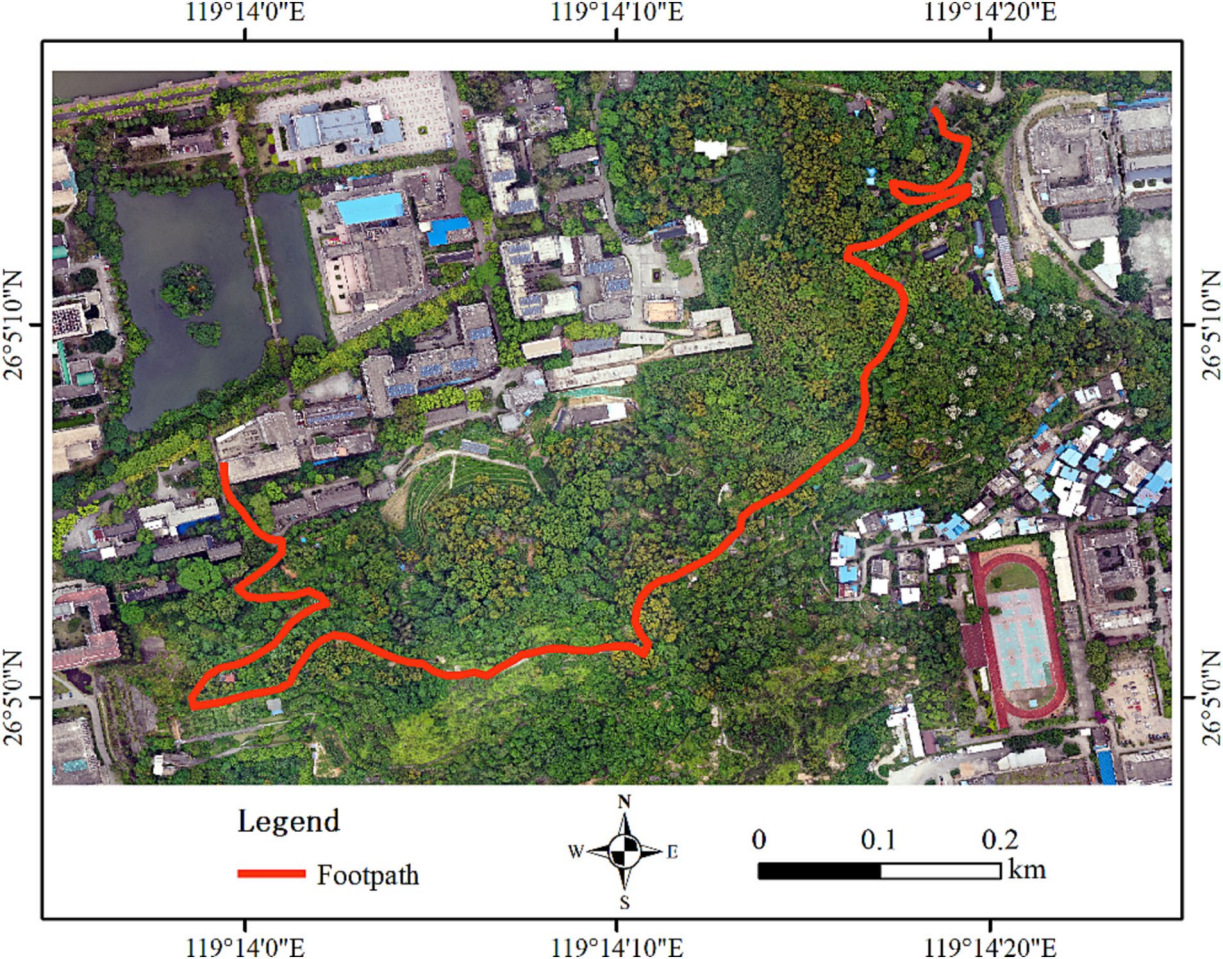
Forest travel activities experience route in Lanyuan forest travel footpath.

The field experiment was conducted in the forested area of the Lanyuan forest, which is inside the university. The location of the field experiment in this research was shown by using the software ArcGIS 10.8. Lanyuan is a typical subtropical laurel forest zone. It is a 10-min walk for FAFU students, who can go there anytime. Despite its proximity to a learning building, this forest footpath is nearly isolated. The study area was a suitable place for conducting forest activities in terms of accessibility, distribution of a variety of vegetation, and gentle slope. The whole distance of the forest footpath is 1.5 km, and the forest travel activities experience route is shown in Figure 1.

Since the environmental situation might influence one’s psychological status, environmental information was collected in this study. The environmental data for the forest travel activities in Lanyuan forest were gathered on December 17, 2019 and presented in Table 2.

**Table 2:** Environmental information of Lanyuan forest travel footpath during the forest travel activities experiment.

Location description	Anion (cm^−3^)	Illumination (LUX)	Carbon dioxide (ppm)	Temperature (°C)	Humidity (%)
Starting point	1,900	238.75	399	16.1	90.5
Lanyuan auditory course	2,970	456.75	437	16.2	96.7
Rest 1	3,390	1377.00	403	15.8	98.9
Visual perception course	2,380	311.50	385	16.3	90.3
Rest 2	4,090	305.50	482	18.5	82.1
Blindfold tactile course	2,500	511.50	381	20.2	75.3
Forest fragrance course	1,850	306.00	387	21.8	64.9
Destination	2,040	416.25	386	19.2	76.1

The forest therapy experiment in this study was operated during the daytime. The illumination was between 238.75–1377.00 LUX. During the forest therapy experiment, the weather was cloudy without rain. The temperature range in the Lanyuan forest travel footpath was about 16–21°C, and the humidity was about 64.9–98.9. Besides, Table 2 shows that the carbon dioxide (CO_2_) was lower on the main footpath (381–482 ppm), and the concentration of anions was about 1,850/cm^3^–4,090/cm^3^. According to the air quality standard in World Health Organization global air quality guidelines (World Health Organization, 2021), the data indicated that the air was between “clean” and “very clean.” Overall, the environmental situation was comfortable for the participants.

### 2.3 Study materials

The study materials to detect environmental information included an anion concentration detector (type: KEC900+, provided by I-Tse Co., Shanghai), CO_2_, temperature, and humidity detector (type: 77535, provided by I-Tse Co., Shanghai), and a digital illumination detector (type: DT1332A, provided by I-Tse Co., Shanghai).

There were both physiological and psychological tests adopted in this study. The physiological material is the Heart Rate Variability (HRV) dynamic electrocardiogram (type: XAB-M3AG, provided by Yocaly Co., Shanghai). This device could collect three-lead electrocardiography (ECG) from the ventricle and transfer the self-calculated data to an Excel spreadsheet. The data could show information on pulse, standard deviation of the Average NN intervals (SDANN), Low-Frequency (LF), High-Frequency (HF), and Low-Frequency/High-Frequency (LF/HF) ratios. The lower pulse rate and SDANN indicate lower stress levels (Li et al., 2008; Rosenberg et al., 2017; Chen et al., 2020), while the lower LF/HF ratio indicates lower stress levels (Malik, 1996; Reed et al., 2005; Rosenberg et al., 2017).

The psychological tests included the Brief Profile of Mood State (BPOMS) questionnaire and the State–Trait Anxiety Inventory (STAI-S) questionnaire (Yu et al., 2017; Furuyashiki et al., 2019; Lee et al., 2019). Both BPOMS and STAI-S questionnaires were used in the Chinese version. In detail, the BPOMS questionnaire includes six subscales: tension-anxiety, depression-dejection, anger-hostility, fatigue-inertia, confusion-bewilderment, and vigor-activity. Each subscale in this BPOMS questionnaire includes five items. The BPOMS questionnaire was translated by Chen et al. (2002), which had an alpha coefficient of six mood states ranging from 0.98 to 0.99 (Chen et al., 2002). In there, the Cronbach’s alpha coefficient for each mood state, including Tension-anxiety, Depression-dejection, and Fatigue-inertia, is 0.99. For other mood states, including Anger-hostility, Vigor-activity, and Confusion-bewilderment, the Cronbach’s alpha coefficient are 0.98. The STAI-S questionnaire includes 20 items, which were translated by Shek which had an alpha coefficient of 0.90 (Shek, 1988). Lower scores on specific items within BPOMS and STAI-S questionnaires are linked to lower stress levels (Yu et al., 2017; Furuyashiki et al., 2019; Lee et al., 2019).

### 2.4 Study procedure

**Figure 2:**
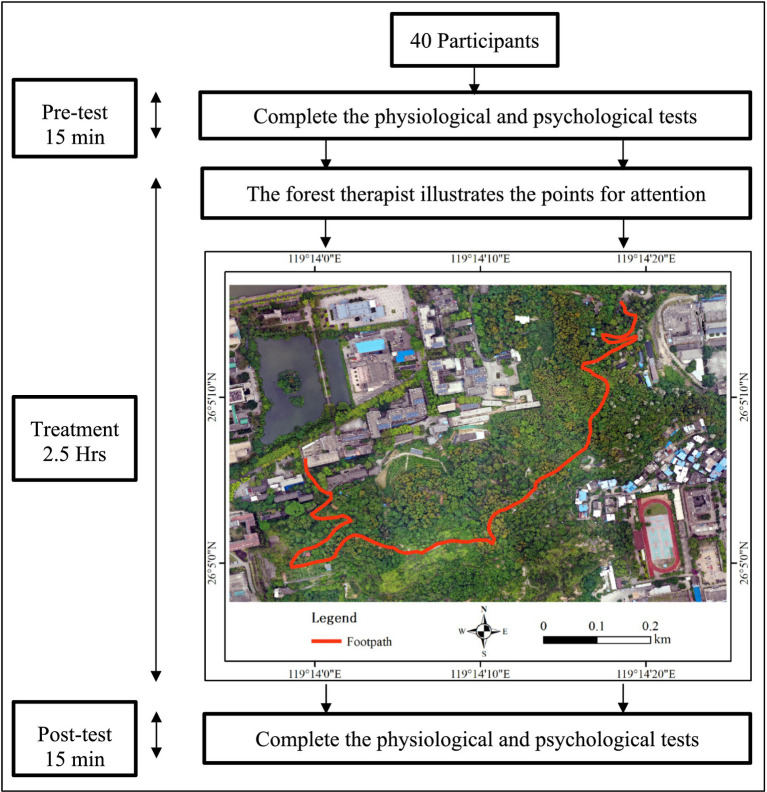
The forest travel activities procedure in this study.

This study investigated the suitable forest locations from July 2018 to February 2019 and confirmed the forest travel activities route in March 2019. Then, participants were recruited from April to November 2019. The experiment was executed on December 7, 2019. Before the forest travel activities experiment, all participants were asked to complete all the tests as the pre-test. Then, a 2.5-h forest travel activities experience was guided by a forest therapist. There were four sessions in the forest travel activities experience which involved “listening to the forest,” “touching trees and clearing the mind,” “seeing the forest by mind vision,” and “forest aromatherapy.” The details for the four sessions of the forest travel activities experience (see Table 3) are based on the references about meditation (Thich and Aitken, 2011; Thich and Katherine, 2017) and forest travel activities (Clifford, 2021), which were revised to be suitable for this study. After the forest travel activities experiment, participants needed to complete all tests as a post-test. The procedure information is given in Figure 2.

**Table 3:** Details of four sessions of forest travel activities.

Period (minutes)	Content of significant activities in forest travel activities
**Session 1: Listening to the forest**	
	Step 1: Make your way into the forest
15	Select a comfortable position Breathe deeply and exhale slowly. Take note of the color, trees, and aroma of the forest at the outset
	Step 2: Walk in the forest
30	Be kind to yourself and stroll; do not hurry Pay attention to everything that comes into your lines of vision, such as the light beaming through the trees, the color of the leaves and flowers, the sounds made by birds, insects, or the wind, the perfume of the trees and land, and the feel and taste of the pure air in the forest
15	Practice walking meditation. Only pay attention to how you walk and how you breathe Concentrate on the present moment while walking; do not concentrate on the past or the future Make the connection between breath and step
	Step 3: Develop a relationship with nature
30	Find a quiet place to sit for at least 20–25 min Maintain silence when observing the natural world around you Use some words to express your current feelings and situation
15	Share your ideas with others while calmly listening to other people’s ideas (do not judge or make noise at this time) Listen to verses for mindful walking
	Step 4: Complete the session
30	Stroll, pay attention to the breath
15	Drink tea and relax Discuss with the forest therapist (optional)
**Session 2: Touching trees and clearing the mind**	
15	Step 1: Make your way into the forest—the same as step 1 (session 1)
45	Step 2: Walk in the forest—the same as step 2 (session 1)
	Step 3: Develop a relationship with nature
30	Find a quiet place to sit for at least 20–25 min Maintain silence, close your eyes, and listen to the sounds in the forest Recognize the sounds you heard in this space
15	Share your ideas with others while calmly listening to other people’s ideas (do not judge or make noise at this time) Listen to verses for our breath
45	Step 4: Complete the session—the same as step 4 (session 1)
**Session 3: Seeing the forest by mind vision**	
15	Step 1: Make your way into the forest—the same as step 1 (session 1)
45	Step 2: Walk in the forest—the same as step 2 (session 1)
	Step 3: Develop a relationship with nature
30	Locate a tree that is both convenient and unlikely to hurt you Gently touch this tree to feel the smoothness or roughness of the stem or leaves Close your eyes and lie back against the tree, hugging it. Put your ear to the stem and listen to the tree’s “breath” (optional) Choose a comfortable activity interaction between you and the tree and hold it for 25 min
15	Share your ideas with others while calmly listening to other people’s ideas (do not judge or make noise at this time) Discuss the importance of forests
45	Step 4: Complete the session—the same as step 4 (session 1)
**Session 4: Forest aromatherapy**	
15	Step 1: Make your way into the forest—the same step 1 (session 1)
45	Step 2: Walk in the forest—the same step 2 (session 1)
	Step 3: Develop a relationship with nature
30	Find a quiet place to sit for at least 20–25 min Maintain silence and pay attention to the breath Recognize the sounds, scents, and sensations inside and outside of you
15	Share your ideas with others while calmly listening to other people’s ideas (do not judge or make noise at this time) Listen to awareness of the body and the breath
45	Step 4: Complete the session—the same as step 4 (session 1)

### 2.5 Data analysis

The statistics from the participants, such as BPOMS, STAI-S, and HRV, were analyzed by SPSS Statistics 27.0. Descriptive statistics comprise mean, standard deviations frequency and percentage to present demographic information of participants and outcome variables. Furthermore, a paired-sample *t*-test (*t*-test) was conducted to compare participants’ physical and psychological responses before and after attending the forest travel activities. All statistical tests used a *p*-value of < 0.05 as the significance level. Additionally, Cohen’s d also was calculated for each dimension to measure the size of the effect of the significant differences between the pre-test and the post-test.

## 3 Results

This study explored the effects of short-term forest travel activities on decreasing university students’ stress levels by analyzing the physiological data (HRV) and the psychological data (BPOMS and STAI-S).

### 3.1 Physiological data

The HRV data could be divided into pulse rate, SDANN, LF, HF, and LF/HF ratios (Li et al., 2008; Rosenberg et al., 2017). The pulse rate indicated the wave frequency of blood flooding. A lower pulse rate reflects lower stress levels (Rosenberg et al., 2017; Chen et al., 2020). The SDANN is the standard deviation of the average NN intervals in around 5 min. The SDANN data could reflect the dysautonomia activity. Lower SDANN data indicated lower stress levels (Chen et al., 2020). Besides, LF and HF are indices that reflect HRV. LF and HF are always opposite; when people feel nervous or stressed, their LF will increase while their HF will decrease, and vice versa (Rosenberg et al., 2017). Previous studies suggested that the LF/HF ratio could be an indicator to detect stress levels, where a lower LF/HF ratio indicates lower stress levels (Malik, 1996; Reed et al., 2005; Rosenberg et al., 2017). This study applied a paired-sample *t*-test to determine the differences between the pre-test and post-test regarding university students’ physiological health on stress affection. The pulse rate, SDANN, LF, HF, and LF/HF ratio data analyses are shown in Table 4.

**Table 4:** Participants’ pre- and post-physiological data.

Physiological indices	Pre-test	Post-test	*t*-value	*p*-value
Pulse rate (bpm)	88.23 ± 7.92	87.95 ± 6.77	0.23	0.823
SDANN (ms)	34.91 ± 15.43	24.91 ± 7.21	3.31	0.003**
LF (ms^2^)	767.32 ± 319.21	756.27 ± 318.69	0.16	0.877
HF (ms^2^)	210.91 ± 169.87	320.91 ± 163.74	−2.54	0.019*
LF/HF	3.10 ± 2.06	2.67 ± 2.46	2.14	0.045*

The results in Table 4 illustrate that the participants showed significantly lower SDANN in the post-test (*M* = 24.91, SD = 7.21) than in the pre-test test (*M* = 34.91, SD = 15.43) with *t* (40) = 3.31, *p* < 0.01. Moreover, the participants presented significantly lower LF/HF ratios in the post-test (*M* = 2.67, SD = 2.46) than in the pre-test (*M* = 3.10, SD = 2.06) with *t* (40) = 2.14, *p* < 0.05. Although the participants’ pulse rate and LF data did not reach significant differences between the post-test score and the pre-test score (*t* = 0.23 and *t* = 0.16, respectively; *p* > 0.05), they all decreased slightly from the pre-test to the post-test. These data meant that the forest travel activities had a positive effect on the stress response of university students in this study, which helped decrease their stress levels.

**Figure 3:**
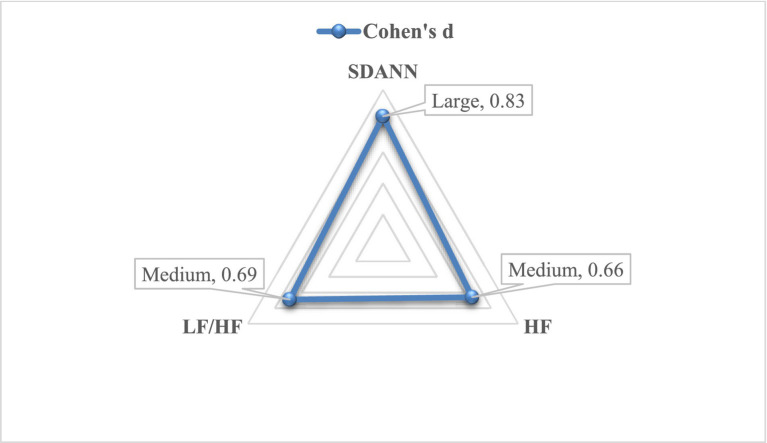
The effect size of the short forest travel activities on physiological indices.

Besides the significant differences, this study also calculated how big those differences were by Cohen’s d (see Figure 3).

Regarding the effect sizes, Figure 3 shows that Cohen’s d of physiological indices had good effect sizes, ranging from 0.66 to 0.83. Noticeably, the average SDANN score received in the post-test was 0.83 standard deviations lower than the average SDANN score received in the pre-test (Cohen’s d = 0.83), which had a large effect on decreasing students’ stress levels. Following this tendency, the effect sizes of HF and LF/HF were medium at 0.66 and 0.69, respectively. These results additionally confirmed the positive effect of short forest travel activities on the nervous or stress levels of university students.

### 3.2 Psychological data

The psychological data could be divided into BPOMS and STAI-S. The BPOMS variable includes six subscales, in which the lower scores mean lower stress levels. The STAI-S variable indicated the level of anxiety (Furuyashiki et al., 2019). Lower STAI-S data means lower stress levels (Furuyashiki et al., 2019). This study employed a paired-sample *t*-test to examine the differences in university students’ psychological health related to stress between the pre-test and post-test phases. The results from the BPOMS and STAI-S questionnaires are shown in Table 5.

**Table 5:** Participants’ pre- and post-psychological data.

Variables	Subscales	Pre-test	Post-test	*t*-value	*p*-value
Emotional states (BPOMS)	Tension-anxiety	2.95 ± 2.93	1.74 ± 2.37	2.09	0.040*
	Depression-dejection	2.48 ± 2.94	1.19 ± 2.43	2.18	0.032*
	Anger-hostility	5.81 ± 3.45	3.60 ± 3.50	2.92	0.004**
	Fatigue-inertia	3.12 ± 3.60	1.60 ± 2.63	2.22	0.029*
	Confusion-bewilderment	3.95 ± 2.78	2.45 ± 2.46	2.62	0.010*
	Vigor-activity	9.88 ± 2.74	11.90 ± 4.76	−2.39	0.020*
Anxiety (STAI-S)	State-anxiety	40.86 ± 7.44	34.21 ± 8.77	3.74	0.000***

The results of Table 5 show that almost all data from all domains of the BPOMS and STAI-S questionnaires were significantly different from the pre-test to the post-test. In more detail, five in six subscales of the BPOMS data (tension-anxiety, depression-dejection; anger-hostility; fatigue-inertia; confusion-bewilderment) showed significantly lower scores in the post-test (*M* = 1.19 ~ 3.60, SD = 2.37 ~ 3.50) than in the pre-test (*M* = 2.48 ~ 5.81, SD = 2.93 ~ 3.60). Additionally, the *t*-values ranged from 2.09 to 2.92 with 0.001 < *p* < 0.05, which meant a statistically significant decrease between the post-test and pre-test regarding the students’ stress levels. In contrast, the only data that showed a significant increase in the post-test (*M* = 11.90, SD = 4.76) compared to the pre-test (*M* = 9.88, SD = 2.74) is the vigor-activity subscale.

**Figure 4:**
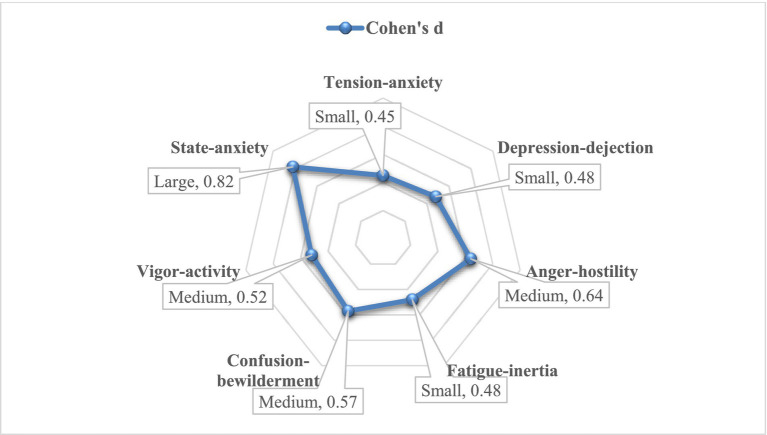
The effect size of the short forest travel activities on psychological indices.

In terms of the STAI-S data, Table 5 shows that participants scored significantly lower State-anxiety in the post-test (*M* = 34.21, SD = 8.77) than in the pre-test (*M* = 40.86, SD = 7.44) with *t* (40) = 3.14, *p* < 0.001. These results meant that the short forest travel activities had a positive effect on the psychological health of university students in this study, which helped decrease their stress levels. Besides the significant changes in the psychological data, this study also showed how big those changes were by Cohen’s d (see Figure 4).

The results in Figure 4 showed that Cohen’s d of psychological indices had quite good effect sizes, ranging from 0.45 to 0.82. However, the STAI-S effect could be stronger than the BPOMS effect on university students’ stress affection. In particular, the effect size of STAI-S data was large at 0.82, while the effect sizes of six subscales in BPOMS data were small and medium levels with Cohen’s d ranging from 0.45 to 0.64. Although the different levels of effect sizes, these results presented the positive effect of forest travel activities on the stress affection of university students.

## 4 Discussion

This study explored the impact of short-term forest travel activities on university students’ stress, employing a comprehensive analysis of physiological and psychological indicators. The results indicate a noteworthy reduction in stress levels among participants, offering insights into the potential benefits of nature-based interventions for university students.

The physiological data, including HRV indices such as SDANN, LF, HF, LF/HF ratio, and pulse rate, consistently exhibited a trend toward reduced stress levels among participants. The significant differences in SDANN and LF/HF ratio indices indicate a positive physiological response to forest travel activities, aligning with previous research emphasizing the relaxing effects of natural environments (Malik, 1996; Reed et al., 2005; Mao et al., 2012a,b; Rosenberg et al., 2017). Although a lower pulse rate is a sign of relaxation, exercise or emotions could easily influence it. Therefore, the accuracy of the pulse rate index should warrant cautious interpretation (Shi et al., 2017; Blasé and Waning, 2019). Moreover, the psychological data, including BPOMS and STAI-S, complement the physiological results by providing insights into the participants’ emotional and mental states. The significant reductions in tension-anxiety, depression-dejection, anger-hostility, fatigue-inertia, confusion-bewilderment, and state-anxiety, along with an increase in vigor-activity, point toward a notable improvement in overall psychological health among the participants. This is similar to previous research demonstrating that walking in natural environments such as natural forests, forests in urban areas, or on-campus helps reduce stress (Mao et al., 2012a,b; Norimasa et al., 2014; Jamali et al., 2020).

Our findings resonate with prior studies that have demonstrated the stress-reducing potential of forest travel activities (Li et al., 2021). However, most previous studies found that forest travel activities could decrease the stress levels of elderly people (Malik, 1996; Yu et al., 2017) and adults (18 years or older) with pre-hypertension or hypertension (Yau and Loke, 2020). Notably, the effects observed in this study are pertinent to a younger demographic—university students—extending the applicability of forest travel activities to a population commonly exposed to academic pressures and mental health challenges (Malik, 1996; Furuyashiki et al., 2019). In our investigation involving university students, we observed a modest 1% decrease in post-test heart rate, accompanied by a notable 50% increase in HF power compared to the pre-test. Conversely, studies conducted with elderly participants in Finland and Japan revealed more remarkable changes, indicating a higher reduction in heart rates (from 3.5 to 5.4%) and a twofold increase in HF power during forest walking and viewing (Yau and Loke, 2020). Moreover, our study showed significant reductions in some mood states, along with an increase in vigor-activity among university students, aligning with previous research involving elderly participants (Yu et al., 2017). However, the Cohen’s d effect sizes of the changes in our study appear to be less than those in a previous study on the effects of forest travel activities on the elderly (ranging from 0.68 to 1.08) (Yu et al., 2017), with ours ranging from 0.45 to 0.82. While both age groups experienced positive outcomes, these variations suggest potential differences in the benefits of forest travel activities between younger and older populations.

The findings presented in this study have important implications, particularly in the context of university students’ health. University life is often characterized by high levels of academic pressure, social demands, and mental health challenges (Craggs, 2012; Downs and Eisenberg, 2012; Schwartz et al., 2021). The positive effects observed in both physiological and psychological data suggest that short-term forest travel activities can serve as an effective intervention for mitigating stress among university students.

The practical applications of these findings are twofold. Firstly, universities and educational institutions can consider integrating short-term forest travel activities into their student well-being programs. These activities, even when conducted over a brief period, have demonstrated the potential to alleviate stress and improve mental and physiological health. Such interventions could be particularly beneficial during stressful academic periods or as part of a broader strategy to support student mental health. Secondly, policymakers and urban planners should take note of the potential benefits of preserving or creating urban green spaces and forests. These natural environments can serve as accessible and cost-effective tools for promoting mental and physiological health among diverse populations, including university students.

However, it is important to acknowledge the study’s limitations. Since different forests might produce different concentrations of anions and varying components of phytoncides, the inferences drawn from the results should be made carefully. Therefore, it is necessary to carry out the following studies on the forests with different properties to test variables of forest properties affecting stress affection through forest travel activities. Moreover, to enhance the robustness of future studies, it is crucial to implement controls for factors such as individual variations in stress susceptibility, pre-existing health conditions, and lifestyle factors. Especially, because women were more drawn to self-care experiential activities like forest travel than men (McEwan et al., 2021), future research should consider incorporating gender-specific controls to better understand potential variations in responses to forest interventions. Furthermore, the absence of a control group is also a limitation. The inclusion of a control group would allow for a more comprehensive assessment of the unique contributions of the forest traveling activities. This would help isolate the effects of natural exposure from other potential influencing variables. Additionally, while some indices demonstrated substantial effect sizes, others yielded smaller or medium effects, suggesting the need for larger sample sizes in future studies. Especially, the effectiveness of mindfulness exercises and the influence of the forest environment remain uncertain. Lastly, as each physiological material has its own mechanical limitations, it is also a kind of limitation of this study.

## 5 Conclusion

In this study, we investigated the impact of short-term forest travel activities on reducing stress levels among university students through a comprehensive analysis of physiological and psychological data. The primary findings of this study clearly demonstrate the substantial positive impact of short-term forest travel activities on reducing stress levels among university students. Both physiological measures, encompassing HRV indices and pulse rate, and psychological assessments, including mood and anxiety scales, consistently showed significant improvements post-forest travel.

Our research advances current knowledge by providing evidence of the effectiveness of short-term forest travel activities in reducing stress among university students. This study contributes to the literature on the benefits of nature exposure, particularly among younger populations. In the broader context, this study not only confirms the positive impact of short-term forest travel activities on university students’ stress levels but also highlights the practical applications of this knowledge. It encourages the integration of nature-based interventions in university well-being programs and underscores the significance of green spaces in urban planning. By bridging the gap between research and practice, this study contributes to the improvement of student’s health and more sustainable cities. It is our hope that these findings will inspire further research and policy changes that prioritize the health of individuals and communities through their connection with the natural world.

## Data availability statement

The raw data supporting the conclusions of this article will be made available by the authors, without undue reservation.

## Ethics statement

The studies involving humans were approved by Fujian Agriculture and Forestry University Human Research Ethics Committee. The studies were conducted in accordance with the local legislation and institutional requirements. The participants provided their written informed consent to participate in this study.

## Author contributions

This study was planned by W-YC, XW, and C-FH. The study was conceptualized and designed by W-YC, L-H-PN, N-HT, H-CW, and C-FH. The collection and analysis of data was organized by XW, S-JY, D-SG, and H-ZL. The first draft of the manuscript was written by W-YC. All authors reviewed and developed to previous versions of the manuscript. All authors contributed to the article and approved the submitted version.

## References

[ref1] Antonelli M. Donelli D. Carlone L. Maggini V. Firenzuoli F. Bedeschi E. (2021). Effects of forest bathing (shinrin-yoku) on individual well-being: an umbrella review. Int. J. Environ. Health Res. 32, 1842–1867. doi: 10.1080/09603123.2021.1919293, PMID: 33910423

[ref2] Bielinis E. Bielinis L. Krupińska-Szeluga S. Łukowski A. Takayama N. (2019). The effect of a short forest recreation program on physiological and psychological relaxation in young polish adults. Forests 10:34. doi: 10.3390/f10010034

[ref3] Bielinis E. Takayama N. Boiko S. Omelan A. Bielinis L. (2018). The effect of winter forest bathing on psychological relaxation of young polish adults. Urban For. Urban Green. 29, 276–283. doi: 10.1016/j.ufug.2017.12.006

[ref4] Blasé K. L. Waning A. V. (2019). Heart rate variability, cortisol and attention focus during shamatha quiescence meditation. Appl. Psychophysiol. 44, 331–342. doi: 10.1007/s10484-019-09448-w, PMID: 31485894

[ref5] Cavallo P. Carpinelli L. Savarese G. (2016). Perceived stress and bruxism in university students. BMC. Res. Notes 9, 514–516. doi: 10.1186/s13104-016-2311-0, PMID: 28003024 PMC5178076

[ref6] Chen K. M. Snyder M. Krichbaum K. (2002). Translation and equivalence: the profile of mood states short form in English and Chinese. Int. J. Nurs. Stud. 39, 619–624. doi: 10.1016/S0020-7489(01)00068-2, PMID: 12100873

[ref7] Chen H. T. Yu C. P. Lee H. Y. (2018). The effects of forest bathing on stress recovery: evidence from middle-aged females of Taiwan. Forests 9:403. doi: 10.3390/f9070403

[ref8] Chen Y. Y. Zhang L. P. Zhang B. Zhan C. A. (2020). Short-term HRV in young adults for momentary assessment of acute mental stress. Biomed. Signal Process Control. 57:101746. doi: 10.1016/j.bspc.2019.101746

[ref9] Cheng X. Liu J. Liu H. Lu S. (2021). A systematic review of evidence of additional health benefits from forest exposure. Landsc. Urban Plan. 212:104123. doi: 10.1016/j.landurbplan.2021.10

[ref10] Chun M. H. Chang M. C. Lee S. J. (2017). The effects of forest therapy on depression and anxiety in patients with chronic stroke. Int. J. Neurosci. 127, 199–203. doi: 10.3109/00207454.2016.1170015, PMID: 27033879

[ref11] Clifford M. A. (2021). Forest bathing step-by step: An optimal flow of referencing in experience the healing power of nature Forest bathing. Newburyport: Red Wheel.

[ref12] Craggs S. (2012). One-third of McMaster students battle depression: Survey CBC news. Available at: https://www.cbc.ca/news/canada/hamilton/headlines/one-third-of-mcmaster-students-battle-depression-survey1.1200815.

[ref13] Dagnino-Subiabre A. (2022). Resilience to stress and social touch. Curr. Opin. Behav. Sci. 43, 75–79. doi: 10.1016/j.cobeha.2021.08.011, PMID: 35187208 PMC8832873

[ref14] Downs M. F. Eisenberg D. (2012). Help seeking and treatment use among suicidal college students. J. Am. Coll. Heal. 60, 104–114. doi: 10.1080/07448481.2011.619611 22316407

[ref15] Fink G. (2017). Stress: Concepts, definition and history. Reference module in neuroscience and biobehavioral psychology. (San Diego, CA: Elsevier), 549–555.

[ref17] Furuyashiki A. Tabuchi K. Norikoshi K. Kobayashi T. Oriyama S. (2019). A comparative study of the physiological and psychological effects of forest bathing (Shinrin-yoku) on working age people with and without depressive tendencies. Environ. Health Prev. Med. 24, 46–11. doi: 10.1186/s12199-019-0800-1, PMID: 31228960 PMC6589172

[ref18] Gong M. K. Wu J. P. Nan H. L. (2017). An empirical study on the effects of viewing forest on human physical and mental health. J. Beijing Univ. Soc. Sci. 16, 44–51. doi: 10.13931/j.cnki.bjfuss.2017060

[ref19] Hansen M. M. Jones R. Tocchini K. (2017). Shinrin-Yoku (Forest bathing) and nature therapy: a state-of-the-art review. Int. J. Environ. Res. Public Health 14:851. doi: 10.3390/ijerph14080851, PMID: 28788101 PMC5580555

[ref20] Hassan A. Jiang T. Guo L. Jiang M. Y. Liu A. Jiang Z. H.. (2018). Effects of walking in bamboo Forest and City environments on brainwave activity in young adults. Evid. Based Complement. Altern. Med. 2018, 1–9. doi: 10.1155/2018/9653857, PMID: 29785198 PMC5896408

[ref21] Hauru K. Lehvävirta S. Korpela K. Kotze D. J. (2012). Closure of view to the urban matrix has positive effects on perceived restorativeness in urban forests in Helsinki, Finland. Landsc. Urban Plan. 107, 361–369. doi: 10.1016/j.landurbplan.2012.07.002

[ref22] Ikei H. Song C. Kagawa T. Miyazaki Y. (2014). Physiological and psychological effects of viewing forest landscapes in a seated position in one-day forest therapy experimental model. Nihon Eiseigaku Zasshi 69, 104–110. doi: 10.1265/jjh.69.104, PMID: 24858505

[ref23] Inoue N. (2014). Stress and atherosclerotic cardiovascular disease. J. Atheroscler. Thromb. 21, 391–401. doi: 10.5551/jat.21709 24561512

[ref24] Jamali T. Kavoosi G. Ardestani S. K. (2020). *In-vitro* and *in-vivo* anti-breast cancer activity of OEO (Oliveria decumbens vent essential oil) through promoting the apoptosis and immunomodulatory effect. J. Ethnopharmacol. 248:112313. doi: 10.1016/j.jep.2019.112313, PMID: 31655147

[ref25] Jiang P. (2023). Research on the current situation of Chinese college students’ test anxiety. Lect. Notes Educ. Psychol. Public Media 23, 226–231. doi: 10.54254/2753-7048/23/20230454

[ref26] Jung W. H. Woo J. M. Ryu J. S. (2015). Effect of a forest therapy program and the forest environment on female workers’ stress. Urban For. Urban Green. 14, 274–281. doi: 10.1016/j.ufug.2015.02.004

[ref27] Kil N. Stein T. V. Holland S. M. Kim J. J. Kim J. Petitte S. (2021). The role of place attachment in recreation experience and outcome preferences among forest bathers. J. Outdoor Recreat. Tour. 35:100410. doi: 10.1016/J.JORT.2021.100410

[ref28] Kim J. G. Jeon J. Shin W. S. (2021). The influence of Forest activities in a university campus Forest on Student's psychological effects. Int. J. Environ. Res. Public Health 18:2457. doi: 10.3390/ijerph18052457, PMID: 33801534 PMC7967591

[ref29] Korpela K. (2003). Negative mood and adult place preference. Environ. Behav. 35, 331–346. doi: 10.1177/0013916503251442

[ref30] Kotera Y. Fido D. (2021). Effects of Shinrin-yoku retreat on mental health: a pilot study in Fukushima, Japan. Int. J. Ment. Health Addict 20, 2652–2664. doi: 10.1007/s11469-021-00538-7

[ref31] Lee H. J. Son Y. H. Kim S. Lee D. K. (2019). Healing experiences of middle-aged women through an urban forest therapy program. Urban For. Urban Green. 38, 383–391. doi: 10.1016/j.ufug.2019.01.017

[ref32] Lee J. Tsunetsugu Y. Takayama N. Park B. J. Li Q. Song C.. (2014). Influence of forest therapy on cardiovascular relaxation in young adults. Evid. Based Complement. Altern. Med. 2014, 1–7. doi: 10.1155/2014/834360, PMID: 24660018 PMC3934621

[ref33] Li Q. S. Liu J. Di Y. B. Xiang H. Liu J. Li B. F. (2008). Observing air anion concentration in Beidaihe and Anion’s evaluation standard. J. Environ. Manag. Coll. China 4, 1–3.

[ref34] Li Q. Morimotoi K. Nakadai A. Inagaki H. Katsumata M. Shimizu T.. (2007). Forest bathing enhances human natural killer activity and expression of anti-cancer proteins. Int. J. Immunopathol. Pharmacol. 20, 3–8. doi: 10.1177/03946320070200s202, PMID: 17903349

[ref35] Li J. Wang G. Wang Z. Wang W. Chen H. He M. (2021). Comparative study of the physiological and psychological effects of forest and urban auditory stimulus on humans. Int. J. Geoheritage Parks 9, 363–373. doi: 10.1016/J.IJGEOP.2021.09.001

[ref36] Lin W. Chen Q. B. Jiang M. G. Zhang X. X. Liu Z. F. Tao J. Y.. (2019). The effect of green space behaviour and per capita area in small urban green spaces on psychophysiological responses. Landsc. Urban Plan. 192:103637. doi: 10.1016/j.landurbplan.2019.103637

[ref37] Liu S. S. Qiao Z. Q. Jin T. W. Wang X. M. Liu X. M. (2018). Scientific research on forest wellness: review and expectation. World For. Res. 31, 26–32. doi: 10.13348/j.cnki.sjlyyj.2018.0053.y

[ref38] Lyu B. Zeng C. Xie S. Li D. Lin W. Li N.. (2019). Benefits of a three-day bamboo forest therapy session on the psychophysiology and immune system responses of male college students. Int. J. Environ. Res. Public Health 16:4991. doi: 10.3390/ijerph16244991, PMID: 31817971 PMC6950568

[ref39] Malik M. (1996). Standards of measurement, physiological interpretation, and clinical use: task force of the European Society of Cardiology and the north American society for pacing and electrophysiology. Circulation 93, 1043–1065. doi: 10.1161/01.CIR.93.5.1043 8598068

[ref40] Mao G. X. Cao Y. B. Lan X. G. He Z. H. Chen Z. M. Wang Y. Z.. (2012a). Therapeutic effect of forest bathing on human hypertension in the elderly. J. Cardiol. 60, 495–502. doi: 10.1016/j.jjcc.2012.08.003, PMID: 22948092

[ref41] Mao G. X. Lan X. G. Cao Y. B. Chen Z. M. He Z. H. Lv Y. D.. (2012b). Effects of short-term Forest bathing on human health in a broad-leaved Evergreen Forest in Zhejiang Province, China. J. Biomed. Environ. Sci. 25, 317–324. doi: 10.3967/0895-3988.2012.03.010 22840583

[ref42] Markwell N. Gladwin T. E. (2020). Shinrin-yoku (forest bathing) reduces stress and increases people’s positive affect and well-being in comparison with its digital counterpart. Ecopsychology 12, 247–256. doi: 10.1089/eco.2019.0071

[ref43] McEwan K. Giles D. Clarke F. J. Kotera Y. Evans G. Terebenina O.. (2021). A pragmatic controlled trial of forest bathing compared with compassionate mind training in the UK: impacts on self-reported wellbeing and heart rate variability. Sustainability 13:1380. doi: 10.3390/su13031380

[ref44] Merabet N. Lucassen P. J. Crielaard L. Stronks K. Quax R. Sloot P. M. A.. (2022). How exposure to chronic stress contributes to the development of type 2 diabetes: a complexity science approach. Front. Neuroendocrinol. 65:100972. doi: 10.1016/j.yfrne.2021.100972, PMID: 34929260

[ref45] Meyera K. Botsch K. (2017). Do forest and health professionals presume that forests offer health benefits, and is cross-sectional cooperation conceivable? Urban For. Urban Green. 27, 127–137. doi: 10.1016/j.ufug.2017.07.002

[ref46] Miriam A. B. Guillermina N. R. Marcela T. S. Nora M. V. (2021). Development and psychometric properties of the adversity and stress scale (ASS): validation in the adult Mexican population. Int. J. Ment. Health Addict. 2021, 1–15. doi: 10.1007/s11469-021-00669-x, PMID: 34720773 PMC8544184

[ref48] Nan H. L. Wang X. P. Chen J. Q. Zhu J. G. Yang X. H. Wen Z. Y. (2013). Forest therapy in Japan and its revelation. World For. Res. 26, 74–78.

[ref49] National Bureau of Statistics of China. (2019). China statistical yearbook 2019 of national bureau of statistics of China. Available at: http://www.stats.gov.cn/english/.

[ref50] Norimasa T. Kalevi K. Juyoung L. Takeshi M. Yuko T. Bum J. P.. (2014). Emotional, restorative and vitalizing effects of forest and urban environments at four sites in Japan. Int. J. Environ. Res. Public Health 11, 7207–7230. doi: 10.3390/ijerph110707207, PMID: 25029496 PMC4113871

[ref51] Oh B. Lee K. J. Zaslawski C. Yeung A. Rosenthal D. Larkey L.. (2017). Health and well-being benefits of spending time in forests: systematic review. Environ. Health Prev. Med. 22, 71–11. doi: 10.1186/s12199-017-0677-9, PMID: 29165173 PMC5664422

[ref52] Park B. J. Tsunetsugu Y. Kasetani T. Kagawa T. Miyazaki Y. (2010). The physiological effects of Shinrin-yoku (taking in the forest atmosphere or forest bathing): evidence from field experiments in 24 forests across, Japan. Environ. Health Prev. Med. 15, 18–26. doi: 10.1007/s12199-009-0086-9, PMID: 19568835 PMC2793346

[ref53] Rajoo K. S. Karam D. S. Abdullah M. Z. (2020). The physiological and psychosocial effects of forest therapy: a systematic review. Urban For. Urban Green. 54:126744. doi: 10.1016/j.ufug.2020.126744

[ref54] Reed M. J. Robertson C. E. Addison P. S. (2005). Heart rate variability measurements and the prediction of ventricular arrhythmias. QJM 98, 87–95. doi: 10.1093/qjmed/hci018 15671474

[ref55] Ribeiro Í. J. S. Pereira R. Freire I. V. de Oliveira B. G. Casotti C. A. Boery E. N. (2018). Stress and quality of life among university students: a systematic literature review. Health Prof. Educ. 4, 70–77. doi: 10.1016/J.HPE.2017.03.002

[ref56] Rosenberg W. V. Chanwimalueang T. Adjei T. Jaffer U. Goverdovsky V. Mandic D. P. (2017). Resolving ambiguities in the LF/HF ratio: LF-HF scatter plots for the categorization of mental and physical stress from HRV. Front. Physiol. 8:360. doi: 10.3389/fphys.2017.00360, PMID: 28659811 PMC5469891

[ref57] Schwartz K. D. Exner-Cortens D. McMorris C. A. Makarenko E. Arnold P. Van Bavel M.. (2021). COVID-19 and student well-being: stress and mental health during return-to-school. Can. J. Sch. Psychol. 36, 166–185. doi: 10.1177/08295735211001653 34040284 PMC8114331

[ref58] Shah M. Hasan S. Malik S. Sreeramareddy C. T. (2010). Perceived stress, sources and severity of stress among medical undergraduates in a Pakistani medical school. BMC Med. Educ. 10:2, 1–8. doi: 10.1186/1472-6920-10-2, PMID: 20078853 PMC2820489

[ref59] Sharma K. Akre S. Chakole S. Wanjari M. B. (2022). Stress-induced diabetes: a review. Cureus 14:e29142. doi: 10.7759/cureus.29142, PMID: 36258973 PMC9561544

[ref60] Shek D. T. (1988). Reliability and factorial structure of the Chinese version of the state-trait anxiety inventory. J. Psychopathol. Behav. Assess. 10, 303–317. doi: 10.1007/BF00960624

[ref61] Shi B. Zhang L. Cao Y. Wang D. N. Zhang W. J. (2017). Comparison study of heart rate variability at resting and real-time motional states. Chinese J. Med. Instrument. 41, 157–160. doi: 10.3969/j.issn.1671-7104.2017.03.001, PMID: 29862757

[ref62] Shin W. S. Shin C. S. Yeoun P. S. (2012). The influence of forest therapy camp on depression in alcoholics. Environ. Health Prev. Med. 17, 73–76. doi: 10.1007/s12199-011-0215-0, PMID: 21503628 PMC3258312

[ref63] Song C. Ikei H. Igarashi M. Takagaki M. Miyazaki Y. (2015). Physiological and psychological effects of a walk in urban parks in fall. Int. J. Environ. Res. Public Health 12, 14216–14228. doi: 10.3390/ijerph121114216, PMID: 26569271 PMC4661642

[ref64] Song C. Ikei H. Miyazaki Y. (2017). Sustained effects of a forest therapy program on the blood pressure of office workers. Urban For. Urban Green. 27, 246–252. doi: 10.1016/j.ufug.2017.08.015

[ref65] Sonntag-Öström E. Nordin M. Dolling A. Lundell Y. Nilsson L. Järvholm L. S. (2015). Can rehabilitation in boreal forests help recovery from exhaustion disorder? The randomised clinical trial forest. Scand. J. For. Res. 30, 732–748. doi: 10.1080/02827581.2015.1046482

[ref66] Thich N. H. Aitken R. (2011). The long road turns to joy: A guide to walking meditation. California: Parallax Press.

[ref67] Thich N. H. Katherine W. (2017). Happy teachers change the world. California: Parallax Press.

[ref68] Tsunetsugu Y. Park B. J. Ishii H. Hirano H. Kagawa T. Miyazaki Y. (2007). Physiological effects of Shinrin-yoku (taking in the atmosphere of the forest) in an old-growth broadleaf forest in Yamagata prefecture, Japan. J. Physiol. Anthropol. 26, 135–142. doi: 10.2114/jpa2.26.135, PMID: 17435356

[ref69] Tsunetsugu Y. Park B. J. Miyazaki Y. (2010). Trends in research related to “Shinrin-yoku” (taking in the forest atmosphere or forest bathing) in Japan. Environ. Health Prev. Med. 15, 27–37. doi: 10.1007/s12199-009-0091-z, PMID: 19585091 PMC2793347

[ref70] Wen L. Y. Shi L. X. Zhu L. J. Zhou M. J. Hua L. Jin Y. L.. (2022). Associations between Chinese college students’ anxiety and depression: a chain mediation analysis. PLoS One 17:e0268773. doi: 10.1371/journal.pone.0268773, PMID: 35653383 PMC9162318

[ref71] World Health Organization. (2021). WHO global air quality guidelines: Particulate matter (PM2.5 and PM10), ozone, nitrogen dioxide, sulfur dioxide and carbon monoxide. Available at: https://www.who.int/publications-detail-redirect/9789240034228. 34662007

[ref72] Yau K. K. Y. Loke A. Y. (2020). Effects of forest bathing on pre-hypertensive and hypertensive adults: a review of the literature. Environ. Health Prev. Med. 25:23. doi: 10.1186/s12199-020-00856-7, PMID: 32571202 PMC7310560

[ref73] Yoshinori O. Noriyuki Y. Shigeru T. (1998). Shinrin-yoku (forest-air bathing and walking) effectively decreases blood glucose levels in diabetic patients. Int. J. Biometeorol. 41, 125–127. doi: 10.1007/s004840050064, PMID: 9531856

[ref74] Yu C. P. Chang W. C. Ramanpong J. (2019). Assessing visitors’ memorable tourism experiences (MTES) in forest recreation destination: a case study in Xitou nature education area. Forests 10:636. doi: 10.3390/f10080636

[ref75] Yu C. P. Lin C. M. Tsai M. J. (2017). Effects of short forest bathing program on autonomic nervous system activity and mood states in middle-aged and elderly individuals. Int. J. Environ. Res. Public Health 14:897. doi: 10.3390/ijerph14080897, PMID: 28792445 PMC5579495

[ref76] Zhuo Y. F. Sun Z. C. (2014). Effect of short-term Forest bathing in urban parks on perceived anxiety of young-adults: a pilot study in Guiyang, Southwest China. Chin. Geogr. Sci. 29, 139–150. doi: 10.1007/s11769-018-0987-x

